# JCV-specific cell-based assays for PML risk assessment in lupus and multiple sclerosis patients with and without natalizumab

**DOI:** 10.3389/fneur.2025.1584083

**Published:** 2025-08-04

**Authors:** Qi Wu, Elizabeth A. Mills, Qin Wang, Guangmei Mao, Yang Mao-Draayer

**Affiliations:** ^1^Department of Pharmaceutical Sciences, University of Michigan, Ann Arbor, MI, United States; ^2^Autoimmunity Center of Excellence, University of Michigan Medical School, Ann Arbor, MI, United States; ^3^Alzheimer’s Drug Discovery Foundation, New York, NY, United States; ^4^School of Public Health, University of Michigan, Ann Arbor, MI, United States; ^5^Autoimmunity Center of Excellence, Multiple Sclerosis Center of Excellence, Arthritis and Clinical Immunology Research Program, Oklahoma Medical Research Foundation, Oklahoma City, OK, United States

**Keywords:** PML, DMT, MS, cytotoxicity index, OX40 immunity index, natalizumab (Tysabri), SLE, BCDT

## Abstract

**Background:**

Progressive multifocal leukoencephalopathy (PML) is an opportunistic infection caused by the JC virus and often fatal. Natalizumab is a highly effective therapy for multiple sclerosis (MS) but is linked to a high incidence of PML. The current metrics used to stratify MS patients at risk for PML are incomplete, leading some patients to prematurely discontinue an effective treatment and others to develop PML despite perceived low risk.

**Objective:**

We sought to develop a combination of cell-based assays using peripheral blood which can be used to provide a more comprehensive assessment of immune system function and complement existing PML risk metrics.

**Methods:**

Our assays measure general and JCV specific responses in CD4^+^ and CD8^+^ T cells following antigen stimulation. We examined responses in systemic lupus erythematosus (SLE) and MS patients with and without PML. Our cytotoxicity index (CTI) measures expression of IFNγ and the degranulation marker CD107a on CD8^+^ T cells, while our OX40 immunity index (OII) measures CD4^+^ T cell activation, as determined by OX40 and CD25 co-expression.

**Results and conclusion:**

We find that the combined metrics can be used to assess JCV immunocompetence, which can distinguish patients with and without PML, and could be used to predict and monitor PML patients from diagnosis onward to facilitate timely intervention.

## Introduction

The rise in the number of cases of natalizumab (NTZ)-associated progressive multifocal leukoencephalopathy (PML) diagnosed in recent years has become a major concern in the multiple sclerosis (MS) community (1). PML is a fetal viral infection of the central nervous system (CNS) that results from the damage of glial and/or neural cells infected by the John Cunningham virus (JCV). Since the lytic infection by JCV leads to the loss of myelinating oligodendrocytes, PML can present with symptoms resembling MS relapse, complicating diagnosis. Although the rate of JCV infection in the general population is around 70–90%, PML requires the mutation of the JC virus and typically only manifests in the context of immunosuppression (2).

NTZ, a highly efficacious therapy for treating relapsing–remitting MS (RRMS), produces a localized immunosuppression within the CNS by inhibiting *α*-4 integrin mediated migration of lymphocytes across the blood brain barrier (3). Due to the high risk for PML, many MS patients clinically benefitting from NTZ treatment have switched to other immunomodulatory therapies, such as B cell depleting therapy (BCDT), fingolimod and dimethyl fumarate (DMF), but some patients still went on to develop PML (4, 5). PML may remain a significant concern in the many patients on NTZ that have switched to BCDT (anti-CD20 monoclonal antibody). Cases of PML have already been reported with BCDT (6), and the risk for treatment-experienced patients is unknown. The use of NTZ for over 2 years, prior history of immunosuppression, and the presence of JCV-specific antibodies have been identified as risk factors in NTZ PML cases (7). Each individual factor on its own may help predict risk for PML but would not determine which patients will actually develop PML. Additional metrics are needed to build a more comprehensive risk profile.

Based on the rationale that the loss of productive JCV-specific T-cell activity drives the development of PML, peripheral based assays have been used to examine the function of JCV specific T cells in globally immunocompromised patients with HIV-associated PML and shown to have some predictive merit (8–10). Due to its primary effect on CNS immune capacity, it was initially unclear if these assays were translatable to NTZ-associated PML, but was plausible because NTZ can also affect T cell activation. Other groups have shown evidence of aberrant JCV specific responses in NTZ-associated PML using assays primarily focusing on the production of IFNγ by CD8^+^ and/or CD4^+^ T-cells (11–13). However, the IFNγ response has not always been consistent in these assays, as some PML patients appeared to elicit productive responses. The expression of the T cell exhaustion marker programmed death-1 (PD-1) on JCV specific T-cells has also been proposed as both a marker and therapeutic target for PML (14). However, PD-1 expression appears to be more prevalent in early PML (10) and may not be useful in monitoring patients with chronic PML. The response of the immune system to a particular antigen, such as JCV, is complex, and thus cannot be adequately captured with metrics looking at the expression of a single cell surface protein or cytokine. Therefore, we have developed a novel set of JCV specific immunity indices which examine a broader response profile. These indices may help monitor patients at high risk for developing PML as well as the vitality of the immune system in patients with PML.

## Methods

### Patients and informed consent

Informed content was obtained from patients prior to participation in the study. The study was approved by the University of Michigan Institutional Review Board. All RRMS patients were treated with NTZ (MS #1–5). The primary progressive (PPMS) patient (MS #6) and lupus patient (PML #3) did not have prior exposure to NTZ. The demographic characteristics and clinical data of the patients, including current risk associated metrics of prior disease modifying therapies (DMTs), time on NTZ, absolute lymphocyte count, and JCV serostatus, are described in Supplementary Table S1. Blood samples were collected in Vacutainer™ Sodium Heparin tubes (BD Biosciences) and processed within 24 h following collection.

### CD107a assay

Whole blood from patients was stimulated with or without Phytohemagglutinin (PHA, 15 μg/mL, Sigma L1668), or human myelin basic protein (MBP, 50 μg/mL, Sigma M0689), or JCV VP1 peptide pool (1 μg/mL PepTivator® JCV peptide pool consists of 15-mer sequences with 11 amino acids overlap covering complete human JCV VP1 protein, Miltenyl Biotechnology Inc.) in the presence of 1:1000 diluted Golgistop (BD Biosciences) and 1:100 diluted anti-CD28/anti-CD49d (BD Biosciences), as well as APC-conjugated anti-human CD107a (BD Biosciences) for 6 h at 37°C with 5% CO_2_. Brefeldin A (1 μg/mL, LC laboratory) was included in the last 4 h of incubation. After removing RBC at the end of culture, the remaining cells were stained with fluorochrome-conjugated antibodies (Supplementary Table S2) and analyzed on a BD FACSCantoII.

### OX40 assay

Adapted from a previously published method (15). Whole blood from patients was diluted with equal volume RPMI and stimulated with or without PHA (15 mg/mL Sigma L1668), MBP (50ug/ml, Sigma M0689 used as antigen-specific positive control), or JCV VP1 peptide pool (1 μg/mL PepTivator® JCV VP1 from Miltenyl Biotechnology Inc.) for 48 h with the presence of 1 μg/mL Brefeldin A for the last 4 h at 37°C with 5% CO_2_. After removing RBC at the end of culture, cells were stained with antibodies described in Supplementary Table S2 and analyzed by flow cytometry using BD FACSCanto II. Culture supernatants from OX40 assay were spun 2000 g for 10 min to remove aggregates and stored frozen at -80°C. For batch cytokine analysis, thawed supernatants were analyzed using BD™ Cytometric Bead Array (CBA) Human Th1/Th2/Th17 CBA kit according to manufacture suggested protocol (BD Biosciences, San Diego, CA, USA).

### Principal component analysis (PCA)

To assess if there is clustering of observations, we used principal component (PC) analysis to analyze JCV-OII, JCV-CTI, G-OII, and G-CTI measurements of all patients. PC1 and PC2 that accounted for the first and secondary majority of the variation within patients were identified. PCA analysis was performed in R version 3.4.

## Results

Ascertaining the predictive potential of a particular metric requires the use of a longitudinal study which tracks the same patients over time, however, due to its rarity, this type of study has not been feasible for PML. Therefore, like previous studies, ours is a primarily cross-sectional analysis between patients with and without PML. Though, we do track one PML patient longitudinally from disease onset to remission. We provide proof of principle that our assay can serve as a read-out of ongoing general and JCV-specific responses using whole blood. Our cohort consists of 3 PML patients at different stages of the disease including 2 NTZ-treated RRMS patients and 1 systemic lupus erythematosus (SLE) patient treated with azathioprine, 2 healthy controls (HC), 5 NTZ-treated RRMS patients and 1 glatiramer acetate treated PPMS patient without PML for comparison. Patient demographics including established risk factors are described in Supplementary Table S1.

CSF from the PML patients was sent to the NIH for diagnostic confirmation and mutation analysis. Active forms of PML are defined by the presence of JCV DNA in the CNS, and remission as the absence of CNS JCV at time of sample collection. PML #1 is in stable condition (chronic remission, “cR”) 3 years following diagnosis and has the granule cell neuronopathy (GCN) variant (involving cerebella without other white matter/supratentorial involvement) associated with mutations in the VP1 region of JCV (16). PML #2 had the more common PML associated mutation in the noncoding regulatory region of JCV (17) and succumbed to sepsis 3.5 years following diagnosis with NTZ-associated PML, at which point JCV was detected in brain tissue (reactivation, “rA”) [described in case report (18)]. To confirm that our assays discriminate between patients with and without PML, as opposed to another variable specific to a particular subset of MS patients, we included SLE patient PML #3, who was recently diagnosed with the GCN variant. We collected samples from this patient immediately after confirmation of the presence (active, “A”) and then absence (acute remission, “aR”) of JCV in the CSF via PCR test.

### Evaluation of cytotoxic potential of CD8 + T cells

**Figure 1:**
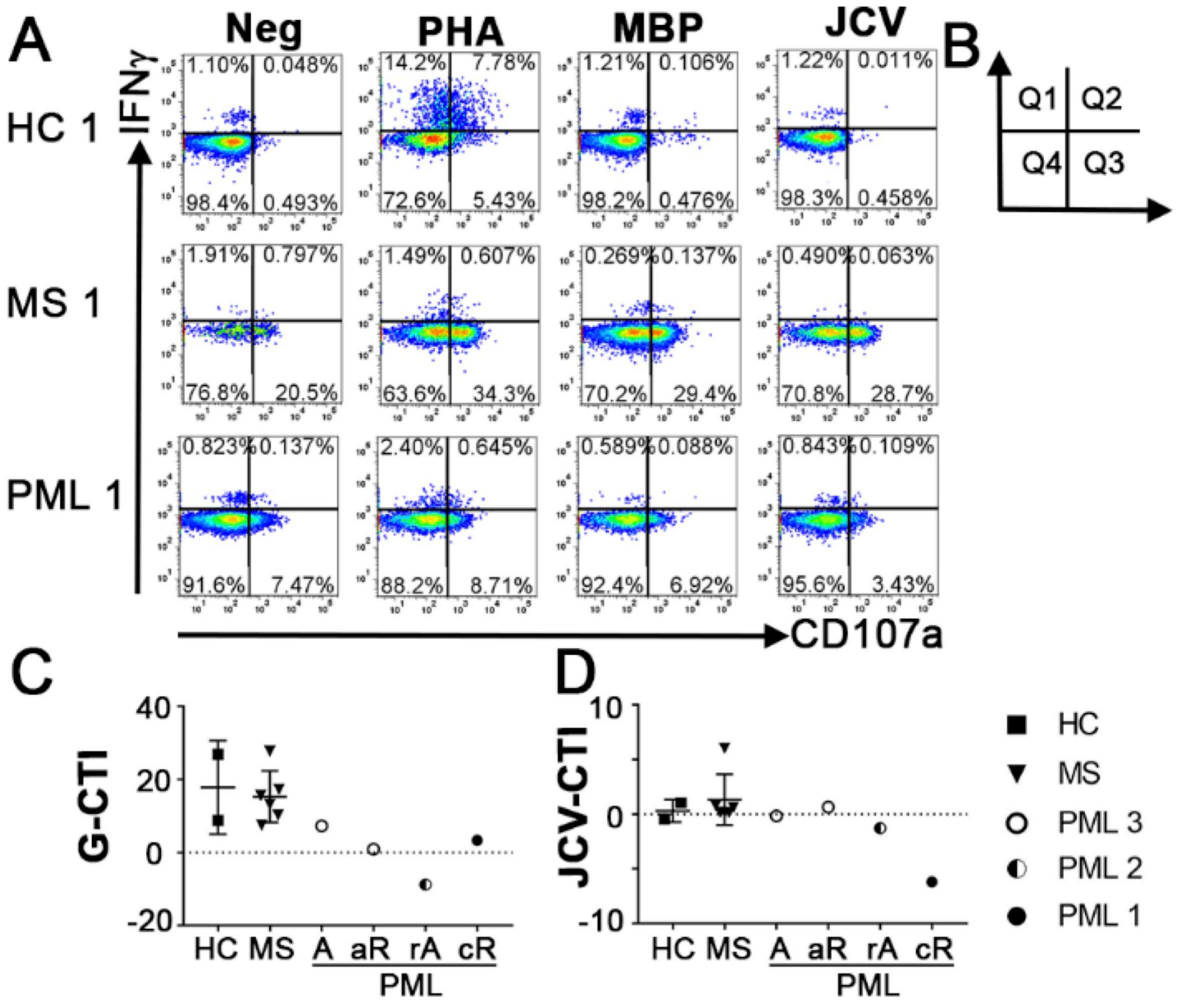
Antigen specific cytotoxicity of CD8^+^ T cells. **(A)** Representative flow cytometry dot plots indicating responses to IFNγ and degranulation marker CD107a following no stimulation (Neg) or stimulation with PHA, MBP, or JCV VP1 peptides in HC, MS patients without PML and MS patients with PML in CD56^−^CD3^+^CD8^+^ T cells. PHA response was used to calculate General Cytotoxicity Index (G-CTI). **(B)** Diagram of dot plot quadrants used in formula for CTI = [(Q1 + Q2 + Q3)_Antigen_−(Q1 + Q2 + Q3)_Neg_]. **(C)** General Cytotoxicity Index (G-CTI) representing general CD8 T cell cytotoxic immune response stimulated by PHA and **(D)** JCV specific CTI (JCV-CTI) representing CD8 T cell cytotoxic response elicited by JCV-derived peptide pool for all tested patients. PML status: A = active, rA = reactivation, aR = acute remission, cR = chronic remission. Shows mean and standard deviation.

Our cytotoxicity index (CTI) considers that cytokine production, including IFNγ, is not the sole indicator of cytotoxic potential, and examines also degranulation using the cell surface marker CD107a (19). Therefore, our index measures CD8^+^ T-cells with cytotoxic potential based on expression of IFNγ and CD107a together or individually [represented by Quadrants (Q) 1–3, as shown in Figures 1A,B], to provide a more thorough and accurate assessment of the antigen specific response. In the calculation of the CTI score, the percentage of antigen stimulated IFNγ and/or CD107a sorted positive cells (Q 1–3) is normalized to the percentage of positive cells (Q1-3) in the unstimulated negative control condition (CTI = [(Q1 + Q2 + Q3)_Antigen_ –(Q1 + Q2 + Q3) _Neg_]). Productive cytotoxic capacity is indicated by a positive number, while anergy is negative. Whole blood is stimulated with either PHA, myelin basic protein (MBP) or JCV peptides. The response to the mitogen PHA provides an indication of general CD8^+^ T-cell mediated cytotoxic activity (G-CTI), while MBP serves as a self-antigen relevant to MS. The use of multiple antigens allows us to determine whether an individual is in a general state of immunosuppression and if the specific response to JCV is compromised.

All MS patients without PML had positive cytotoxic capacity toward all antigens tested (Figure 1; Table 1). In contrast, the PML patients had negative CTI scores toward multiple antigens, including JCV (Figures 1C,D; Table 1). PML patient #3.1 (active) had a modest G-CTI of 7.3, PML #1 (chronic remission) had a weak G-CTI of 3.3, whereas PML #2 (reactivation) had an extremely anergic G-CTI of −8.8, indicating the patient was severely immunocompromised (Table 1), as compared to an average G-CTI of 15.3 ± 7.1 for MS patients without PML (Figure 1C). Notably, in PML #3 the JCV-CTI switched from a negative value of −0.09 in active PML to a positive value of 2.8 during the acute remission period (Figure 1D; Table 1), suggesting that our assay is sensitive to PML status.

**Table 1:** CD107a antigen specific cytotoxicity index in patients with and without PML.

	Neg			PHA			MBP			JCV VP1			Cytotoxicity Index (CTI)		
IFNγ	+	+	−	+	+	−	+	+	−	+	+	−	Gen	MBP	JCV
CD 107a	−	+	+	−	+	+	−	+	+	−	+	+	G-CTI	MBP-CTI	JCV-CTI
HC 1	0.11	0.05	0.49	14.2	7.78	5.53	1.21	0.11	0.48	1.22	0.01	0.46	26.9	1.14	1.04
HC 2	1.58	0.16	0.47	3.17	1.65	6.15	1.37	0.11	1.78	0.66	0.07	1.06	8.76	1.04	−0.42
MS 1	1.91	0.80	20.5	1.49	0.61	34.3	0.27	0.14	29.4	0.49	0.06	28.7	13.2	6.59	6.05
MS 2	0.54	0.03	1.29	21.2	3.30	5.14	0.46	0.09	5.06	0.66	0.03	1.36	27.78	3.74	0.19
MS 3	0.33	0.00	0.04	14.3	0.65	1.03	0.56	0.03	0.05	0.90	0.03	0.07	15.6	0.27	0.63
MS 4	0.00	0.05	0.22	2.71	2.44	5.42	0.13	0.03	0.70	0.01	0.01	0.40	10.3	0.57	0.15
MS 5	0.35	0.00	59.4	0.29	0.88	66.1	0.04	0.10	60.8	0.02	0.01	59.9	7.52	1.19	0.18
MS 6	0.00	0.54	15.4	22.2	2.71	8.32	0.46	0.37	18.3	4.26	1.36	11.1	17.3	3.19	0.78
PML1* cR	0.82	0.14	7.47	2.40	0.66	8.71	0.59	0.09	6.92	0.84	0.11	1.29	3.33	−0.83	−6.19
PML 2 rA	8.75	0.83	2.02	1.30	0.00	1.52	10.7	0.61	1.05	8.10	0.55	1.68	−8.78	0.76	−1.27
PML 3.1 A	0.59	0.04	0.42	1.89	1.76	4.68	0.13	0.06	0.29	0.46	0.03	0.41	7.28	−0.57	−0.15
PML 3.2 aR	3.82	0.00	0.53	0.99	1.24	3.05	1.39	0.14	0.55	4.19	0.19	0.64	0.93	−2.27	0.66

CTI = [(Q1 + Q2 + Q3)_Antigen_ – (Q1 + Q2 + Q3)_Neg_] (see Figure 1). G-CTI: CTI calculated when cells were stimulated with PHA; MBP-CTI: CTI calculated when cells were stimulated with MBP; JCV-CTI: CTI calculated when cells were stimulated with JCV derived peptide. Responses measured in CD56^−^CD3^+^CD8^+^ T cells. *PML status: A = active PML, rA = reactivation, aR = acute remission, cR = chronic remission.

### Evaluation of immune competency of CD4 + T cells

**Figure 2:**
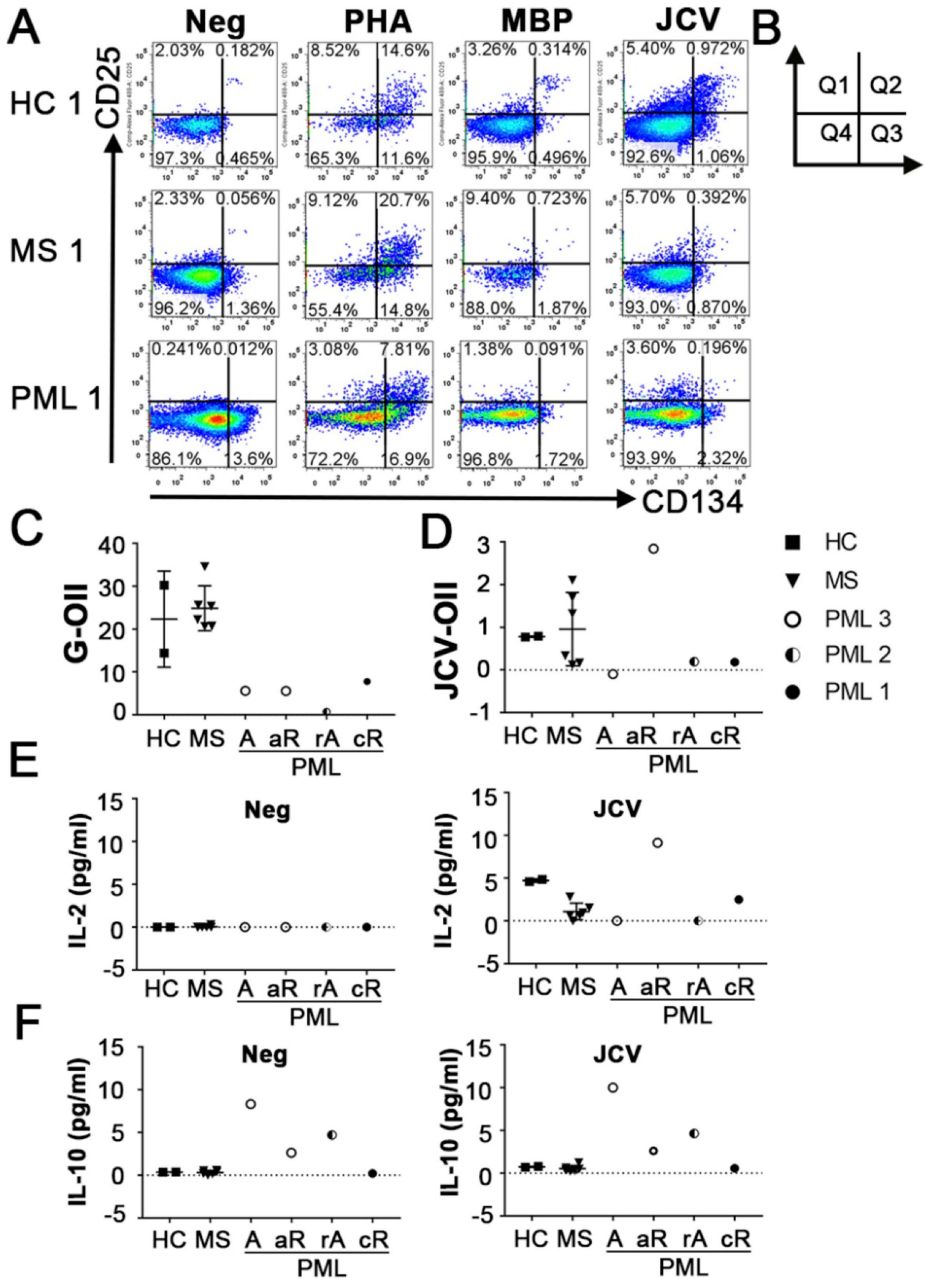
Antigen specific immunity index of CD4^+^ T cells. **(A)** Representative flow cytometry dot plots indicating CD25 and CD134 (OX40) up-regulation following no stimulation (Neg) or stimulation with PHA, MBP, or JCV VP1 peptides in HC, MS patients without PML and MS patients with PML in CD3^+^CD4^+^ T cells. PHA response was used to calculate General OX40 Immunity Index (G-OII). **(B)** Diagram of dot plot quadrants used in formula for OII = [Q2_Antigen_-Q2_Neg_]. **(C)** General OX40 Immunity Index (G-OII) representing CD4 T cell immune response stimulated by PHA and **(D)** JCV specific OII (JCV-OII) representing CD4 T cell response elicited by JCV-derived peptide pool for all tested patients. PML status: A = active, rA = reactivation, aR = acute remission, cR = chronic remission. **(E)** IL-2 levels and **(F)** IL-10 levels in media following no stimulation (Neg) or JCV VP1 peptide stimulation (JCV) for OX40 assay detected using CBA. Values of zero indicate below level of detection for CBA (0.6 pg/mL). Panel **(C–F)** shows mean and standard deviation.

**Figure 3:**
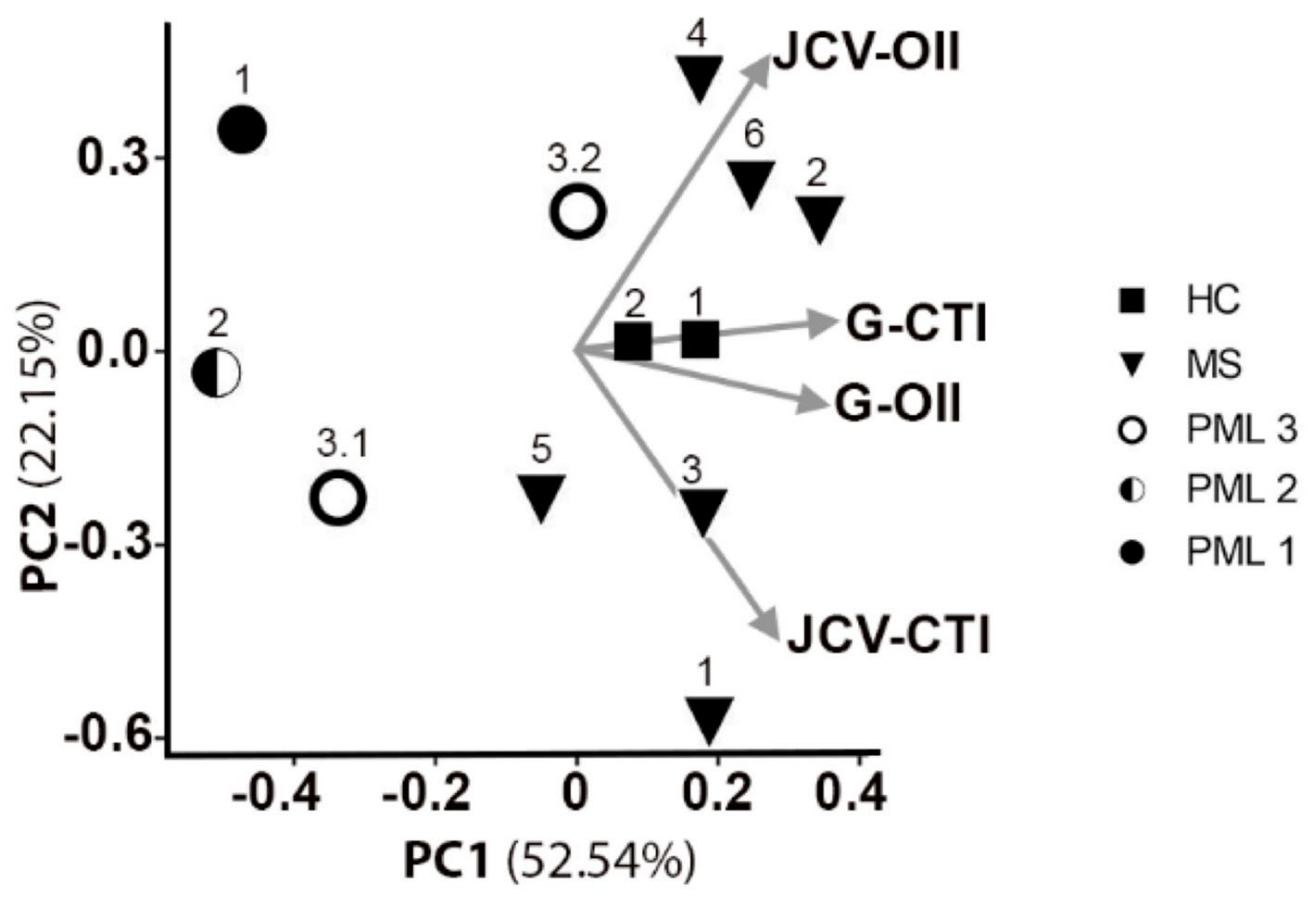
Principal components analysis of general and JCV-specific CTI and OII for predicting PML risk. 2D PCA projection including vectors for G-CTI, G-OII, JCV-CTI, and JCV-OII values for all tested HC, MS, and PML patients. PC1 explains 52.5% of variation in the dataset, while PC2 explains 22.2% of variation. The arrows indicate loading for all 4 measurements. PML patient 3 shown at active (3.1) and acute remission (3.2).

CD4^+^ T cells also play an important role in anti-JCV immunity (20, 21). The OX40 Immunity Index (OII) detects antigen specific CD4^+^ T-cells (15), which assesses immunocompetence. OX40 receptor (CD134) and interleukin-2 receptor alpha chain (CD25) are co-expressed on the surface of activated T-cells, thus their upregulation following stimulation provides evidence for an antigen specific response (22). The OII is calculated by normalizing the percentage of activated CD25^+^CD134^+^ sorted cells (Q2) to the percentage of double-positive sorted cells (Q2) in the unstimulated negative control (OII = [Q2Antigen-Q2Neg]) (Figures 2A,B). We tested our assay by assessing CD4^+^ T cell responses from the same cohort of patients and the same antigens as described for the CTI assay. HC and MS patients without PML demonstrated immunity to all antigens tested, except for negative MBP-OII in two MS patients (Table 2). The average General OX40 Immunity Index (G-OII) value for the PML patients of 6.3 ± 3.0 was lower than for MS patients without PML, 24.9 ± 5.2 (Figure 2C), especially PML #2 (reactivation) with an extremely weak G-OII value of 0.7. Principal components analysis (PCA) considering both general and JCV-specific values for the CTI and OII indices reveals that except in the context of acute remission, the PML patients were separated from other observations, while HC and MS patients were clustered together (Figure 3).

**Table 2:** OX40 antigen specific immunity index in patients with and without PML.

	Neg	PHA	MBP	JCV	Immunity index (OII)		
CD134	+	+	+	+	General	MBP	JCV
CD25	+	+	+	+	G-OII	MBP-OII	JCV-OII
HC 1	0.18	14.60	0.31	0.97	14.42	0.13	0.79
HC 2	0.24	30.50	0.66	1.01	30.26	0.42	0.77
MS 1	0.06	20.70	0.72	0.39	20.64	0.67	0.34
MS 2	0.20	25.80	0.44	1.52	25.56	0.24	1.32
MS 3	0.02	34.60	0.51	0.19	34.58	0.49	0.17
MS 4	0.46	21.20	0.75	2.56	20.74	0.29	2.10
MS 5	0.40	25.80	0.39	0.52	25.40	−0.01	0.12
MS 6	0.74	23.00	0.44	2.44	22.26	−0.30	1.70
PML 1 cR*	0.01	7.81	0.09	0.20	7.80	0.08	0.18
PML 2 rA	0.67	1.37	0.28	0.87	0.70	−0.39	0.20
PML3.1 A	0.35	5.96	0.24	0.26	5.61	−0.11	−0.09
PML3.2 aR	0.54	6.13	1.56	3.38	5.59	1.02	2.84

OX40 Immunity Index OII = [Q2_Antigen_−Q2_Neg_] (see Figure 2). G-OII: OII calculated when cells were stimulated with PHA; MBP-OII: OII calculated when cells were stimulated with MBP; JCV-OII: OII calculated when cells were stimulated with JCV derived peptide pool. Responses measured in CD3^+^CD4^+^ T cells. *PML status: A = active, rA = reactivated, aR = acute remission, cR = chronic remission.

We also measured the level of secreted cytokines in the absence of stimulation (Neg) and following JCV antigen stimulation in our OX40 assay using a cytometric bead array (CBA). Only individuals without evidence of an active form of PML (defined as detectable JCV in CNS) had IL-2 levels above the limit of detection following stimulation with JCV (Figure 2E). The lack of (detectable) IL-2 in patients with active (active/reactivated) PML may stem from the increased levels of IL-10, an inhibitor of IL-2 production (23), both at baseline and in response to JCV in these patients (Figure 2F). This suggests IL-10 production is a relevant risk factor and is consistent with a previous report indicating elevated CSF IL-10 levels and induction of a JCV-specific IL-10 response in CD4^+^ T cells in NTZ-associated PML patients (13).

## Discussion

Our peripheral blood cell-based assays provide an indication of a patient’s overall and JCV specific immune competence, which can be combined with other established risk related metrics, such as patient age and JCV serostatus, to create a more comprehensive risk assessment for PML. The PML patients demonstrated lower overall CD8^+^ cytotoxic capacity and CD4^+^ T-cell activation capacity, suggesting that decreased immune function is a major risk factor for PML. We found that our CTI was sensitive to PML status, and may be useful in the assessment of remission, as patient PML#3 developed a positive JCV-CTI during a period of acute clinical remission. We will continue to monitor this patient to determine whether immune exhaustion to JCV emerges in the chronic remission period, as we found in the other patients with chronic PML.

The transition from a negative (active) to a positive (acute remission) JCV-OII for PML #3 coupled with the positive JCV-OII values for those with chronic PML (Figure 2D; Table 2) suggests that the development of CD4^+^ T cell mediated immunity to JCV may be important for prolonged survival in patients with PML, however, it will be necessary to do longer term follow-up to make this determination. Similarly, a decrease in G-OII values over time may indicate that a patient’s immune responsiveness has been compromised, which could lead to the reactivation of JCV or another opportunistic infection, such as occurred in PML #2 with the extremely weak G-OII response and eventual fatal outcome. Therefore, the continued monitoring of PML survivors may be necessary for long-term disease management. In order to demonstrate the prognostic value of our assays along these measures, it will be necessary to assay additional newly diagnosed PML patients and continue to perform follow-up analyses over time in relation to clinical outcome. Our assays also could be used to improve the clinical efficacy of immunotherapy in PML patients in testing the antigen specific responsiveness of the *ex vivo* expanded JCV specific T cells prior to and after adoptive transfer, and correlate with the amelioration of disease (7).

In terms of building a predictive risk profile, multiple factors need to be considered. The combination of the CTI and OII indices provides an informative assessment of a patient’s overall immune response to JCV, which cannot be captured by a single metric. Based on this cohort, we estimate that having at least one negative component places patients at higher risk and necessitates frequent monitoring, while the greatest risk is associated with negative values along both component axes. Notably, our indices measuring JCV-specific functional immune responses did not correlate with JCV antibody titer (see Supplementary Table S1), as antibody titer reflects past exposure to JCV, while the CTI and OII indicate current immune competency toward JC. Therefore, the use of our cell-based assay may be useful in further stratifying patients that are found to be JCV seropositive. Ultimately, however, the JCV-specific response is only one of several PML risk associated measures and will need to be combined with other factors such as medical history and genetic factors, including the JCV variant(s) residing within an individual, in order to develop a truly predictive assessment (23). A limitation of our study is small sample size. Given the rarity of PML, it is not feasible to follow patients longitudinally before and after development of PML. We put forward our cross-sectional analysis of patients with and without the disease as proof of principle. Future multi-centered longitudinal studies following high risk patients may provide more information. Though analysis of more patients is needed for future studies, the results of this initial study show promise toward the goal of improved prediction, prevention, and management of JCV-associated diseases. Our assays could potentially be widely used in predicting other DMT associated PML as well as in lupus and other etiologies to assess the risk of developing PML (24, 25). DMTs such as BCDT and S1P modulators underestimate JCV index value (26); using our assays to measure CD4 and CD8 cell-based immunity will allow one to more accurately predict PML risk and monitor for treatment response. Recent studies also showed that higher prevalence of anti-JCV Ab seropositivity in East Asia ranging 69.5 to 80% (27–29), whereas the worldwide prevalence is approximately 57% (30, 31). Future studies should also include various ethnicities across different geographic areas.

## Data Availability

The original contributions presented in the study are included in the article/supplementary material, further inquiries can be directed to the corresponding author.
