# Supplementary material for: JCV-specific cell-based assays for PML risk assessment in lupus and multiple sclerosis patients with and without natalizumab
Source: Front Neurol. 2025 Aug 4;16:1584083. doi: 10.3389/fneur.2025.1584083 (PMC12360262; doi:10.3389/fneur.2025.1584083)
Supplement: Supplementary file 1 [file Data_Sheet_1.pdf]

Table SI. Patient Characteristics

Patient	Age	Sex	*Race	MS disease duration (yrs)	Past DMT (in order)	Other DMT duration	NTZ duration	DMT at time of blood collection	WBC Count/ ALC Count (K/µl)	JCV status (index)
HC 1 162	48	M	C	0	N/A	N/A	N/A	N/A	8.0/2.3	Not tested
HC 2 163	52	F	C	0	N/A	N/A	N/A	N/A	4.1/1.4	Not tested
RRMS 1 157	38	M	C	13	Glatiramer acetate, NTZ	2yrs	1.5 yrs	NTZ	6.7/1.1	Positive (0.51)
RRMS 2 154	41	M	C	2	DMF, NTZ	2 yrs	0.75 yr	NTZ	7.8/1.2	Positive (3.32)
RRMS 3 142	54	F	C	14	IFN-β1a, NTZ, Glatiramer acetate, DMF, Teriflunomide, NTZ	5 yrs	6 yrs	NTZ	8.0/3.2	Positive (2.21)
RRMS 4 161	63	M	C	38	Azathioprine, IFN-β1a, NTZ, Fingolimod, NTZ	20 yrs	6 yrs	NTZ	10.7/2.9	Negative (0.10)
RRMS 5 167	41	F	C	13	Teriflunomide, NTZ, Fingolimod, NTZ	1.5 yrs	2.5 yrs	NTZ	7.0/3.0	Positive (0.45)
PPMS 6 153	61	F	C	30	Glatiramer acetate	1 yr	None	None	8.6/2.3	Not tested
PML 1 Chronic remission 145	52	F	C	13	IFN-β1a, NTZ, IFN-β1a, NTZ	2 yrs	9 yrs	None	6.5/1.9	Positive (1.05)
PML 2 reactivation 141	49	M	C	14	IFN-β1a, Mitoxantrone, NTZ	5 yrs	3.5 yrs	None	25.4/0.2	Positive (N/A)
PML 3 acute 170	51	F	C	SLE	Azathioprine and prednisone	8 yrs	None	AZA	6.1/0.6	Positive (3.75)

***C=Caucasian**
