# Supplementary material for: JCV-specific cell-based assays for PML risk assessment in lupus and multiple sclerosis patients with and without natalizumab
Source: Front Neurol. 2025 Aug 4;16:1584083. doi: 10.3389/fneur.2025.1584083 (PMC12360262; doi:10.3389/fneur.2025.1584083)
Supplement: Supplementary file 2 [file Data_Sheet_2.docx]

| **Supplemental Table 2S** | | **Antibodies Used in The Assays** | |  |
| --- | --- | --- | --- | --- |
|  |  |  |  |  |
|  | **Antibodies** | **Clone** | **Vender** | **Cat #** |
|  | **CD107 Assay** |  |  |  |
|  | CD3 PE-Cy7 | UCHT1 | Tonbo | 60-0038-T100 |
|  | CD4 PerCP Cy5.5 | OKT4 | Tonbo | 65-0048-T100 |
|  | CD8 BV510 | SK1 | Biolegend | 344732 |
|  | IFNγ FITC | MKTmAb35 | eBiosciences | 53-7319-41 |
|  | CD107 APC | H4A3 | BD | 641581 |
|  | CD14 APC-Cy7 | MfP9 | BD | 557831 |
|  | **OX40 Assay** | |  |  |
|  | CD25 FITC | BC96 | Biolegend | 302604 |
|  | OX40 PE | Per-ACT35 | Biolegend | 350004 |
|  | CD4 PerCP Cy5.5 | OKT4 | Tonbo | 65-0048-T100 |
|  | CD3 PE-Cy7 | UCHT1 | Tonbo | 60-0038-T100 |
|  | CD14 APC-Cy7 | MfP9 | BD | 557831 |
